# Distinctive Effects of Fullerene C_60_ and Fullerenol C_60_(OH)_24_ Nanoparticles on Histological, Molecular and Behavioral Hallmarks of Alzheimer’s Disease in APPswe/PS1E9 Mice

**DOI:** 10.3390/antiox14070834

**Published:** 2025-07-08

**Authors:** Sholpan Askarova, Kseniia Sitdikova, Aliya Kassenova, Kirill Chaprov, Evgeniy Svirin, Andrey Tsoy, Johannes de Munter, Anna Gorlova, Aleksandr Litavrin, Aleksei Deikin, Andrey Nedorubov, Nurbol Appazov, Allan Kalueff, Anton Chernopiatko, Tatyana Strekalova

**Affiliations:** 1 Center for Life Sciences, National Laboratory Astana, Nazarbayev University, Astana 010000, Kazakhstan; 2 Institute of General Pathology and Pathophysiology, Moscow 125315, Russia; 3 Department of General Biology and Genomics, Faculty of Natural Sciences, L.N. Gumilyov Eurasian National University, Astana 010000, Kazakhstan; 4 Institute of Physiologically Active Compounds at Federal Research Center of Problems of Chemical Physics and Medicinal Chemistry, Russian Academy of Sciences, Chernogolovka 119071, Russia; 5 Neuroplast BV, 6222 NK Maastricht, The Netherlands; 6 Research and Education Resource Center, Peoples Friendship University of Russia, Moscow 117198, Russia; 7 Department of Normal Physiology, Sechenov First Moscow State Medical University, Moscow 119991, Russia; 8 Laboratory of Genetic Technology and Gene Editing for Biomedicine and Veterinary, National Research, Belgorod State University, Belgorod 308015, Russia; 9 Laboratory of Engineering Profile Physical and Chemical Methods of Analysis, Korkyt Ata Kyzylorda University, Kyzylorda 120014, Kazakhstan; 10 Department of Biosciences and Bioinformatics, Suzhou Municipal Key Laboratory of Neurobiology and Cell Signaling, School of Science, Xi’an Jiaotong-Liverpool University, Suzhou 215123, China

**Keywords:** Alzheimer’s disease, antioxidants, fullerene C_60_, fullerenol C_60_(OH)_24_, APPswe/PS1E9 mice

## Abstract

Fullerenes and fullerenols exhibit antioxidant and anti-inflammatory properties, making them promising candidates for Alzheimer’s disease (AD) therapy. Unlike conventional anti-inflammatory drugs, these compounds have multitargeted effects, including their ability to inhibit amyloid fibril formation. However, few studies have explored their efficacy in high-validity AD models. Female APPswe/PS1E9 (APP/PS1) mice and their wild-type (WT) littermates were orally administered with fullerene C_60_ (0.1 mg/kg/day) or fullerenol C_60_(OH)_24_ (0.15 mg/kg/day) for 10 months starting at 2 months of age. Behavioral assessments were performed at 12 months of age. Amyloid plaque density and size were analyzed in the brain regions using Congo red staining. The expression of genes related to inflammation and plasticity was examined, and an in vitro assay was used to test the toxicity of fullerenol and its effect on amyloid β peptide 42 (Aβ42)-induced reactive oxygen species (ROS) production. Fullerenol reduced the maximum plaque size in the cortex and hippocampus, decreased the small plaque density in the hippocampus and thalamus, and prevented an increase in glial fibrillary acidic protein (GFAP) positive cell density in the mutants. Both treatments improved cognitive and emotional behaviors and reduced *Il1β* and increased *Sirt1* expression. In vitro, fullerenol was non-toxic across a range of concentrations and reduced Aβ42-induced ROS production in brain endothelial cells and astrocytes. Long-term administration of fullerene or fullerenol improved behavioral and molecular markers of AD in APP/PS1 mice, with fullerenol showing additional benefits in reducing amyloid burden.

## 1. Introduction

Alzheimer’s disease (AD) is a progressive neurodegenerative disease which affects approximately 10% of people aged 65–75 and 32% of the elderly aged 80 and above [1,2]. The pathophysiology of AD is thought to be driven by the accumulation of amyloid plaques composed of amyloid-beta peptide (Aβ) in the brain, which leads to oxidative stress, mitochondrial dysfunction, enzyme dysregulation, neuroinflammation, and neuronal death. However, most clinical trials aimed at reducing the levels of toxic Aβ aggregates in the brain have failed, and AD remains an incurable disease with several new approaches still in development.

Recent research has highlighted the potential of fullerene-based nanoparticles as anti-aging compounds [3,4] and therapeutic agents for AD [5,6,7,8]. For example, a single administration of water-soluble derivatives of fullerene (1 mg/kg^−1^) improved spatial memory in C57B1/6 mice in an object recognition test [8]. In the scopolamine model of AD-like conditions, subchronic treatment with a colloidal solution of fullerene C_60_ (21 µg/mL, 1 mL) significantly improved spatial memory in the Morris water maze memory test [6]. Several studies have reported the beneficial effects of fullerene C_60_ and its derivatives on intracerebral Aβ infusions [9,10]. Pretreatment with hydrated fullerene C_60_ (C_60_HyFn) restored hippocampal theta and cortical beta electroencephalogram (EEG) rhythms disrupted by infusion of Aβ42 protein in rats [9]; hydrated fullerene C_60_ increased neuronal survival after intracerebral infusions of Aβ25-35 peptide in rats [10]. Finally, Dai et al. used APPswe/PS1E9 (APP/PS1) transgenic mice, a well-established AD model [11], to investigate the effects of subchronically injected fullerene C_60_ suspension (460 μg/kg, i.p.) and found significant effects of the treatment on memory acquisition in the Morris water maze [7].

Fullerenes are molecular forms of carbon in the form of a truncated icosahedron with a mass of 720 amu The most symmetrical and thoroughly studied member of the fullerene family is fullerene C_60_, in which carbon atoms form a truncated icosahedron consisting of 20 hexagons and 12 pentagons. In vitro studies have demonstrated the ability of fullerene nanoparticles to inhibit Aβ aggregation [5,12]. Their hydrophobic surfaces facilitate interactions with Aβ peptides through aromatic stacking mechanisms, disrupting β-sheet formation, and interfering with fibril nucleation and elongation [5]. These interactions, elucidated through molecular dynamics simulations, underlie the molecular basis of their anti-aggregation effects [5]. Furthermore, polyhydroxylated fullerenes are highly effective free-radical scavengers. Several studies have reported their interactions with reactive oxygen species (ROS) [13,14], and they have been proposed as potential anti-aging [3,4] and neuroprotective [15,16,17] antioxidants.

Although fullerene C_60_ is a unique carbon-based nanomaterial with promising biomedical potential, its pharmacokinetic profile is problematic owing to its hydrophobicity, large size, and structural instability [14], and it does not cross the blood-brain barrier (BBB) [18]. Limited data available for the pharmacokinetics and biodistribution of pristine fullerene C_60_ showed that a single oral administration of fullerene C_60_ dissolved in oil resulted in limited bioavailability and slow absorption, indicating delayed uptake. The compound exhibited extensive tissue distribution and moderate elimination, suggesting its accumulation in lipid-rich organs and prolonged systemic retention [4]. Fullerene C_60_ accumulates primarily in the liver, lungs, kidneys, heart, adrenal glands, thymus, and spleen. Notably, no detectable amount of fullerene C_60_ was found in the brain tissue, suggesting limited penetration into this organ [18]. However, seven months of oral administration of C_60_ dissolved in olive oil in repeated doses (1.7 mg/kg/day) almost doubled the lifespan of the experimental animals [4]. Importantly, recent studies revealed marketable effects of the C_60_ on gut microbiome, which was suggested to underlie its antioxidant and anti-inflammatory effects in a model of Parkinson’s disease (PD) [17].

Because fullerene C_60_ is a hydrophobic molecule that is insoluble in water, a number of hydroxylated forms of fullerene containing hydroxyl (–OH) groups on the surface of fullerene molecules have been developed. Among them, fullerenol (C_60_(OH)_24_) was shown to exert the maximal ability to counteract amyloid aggregation [17]. Sun et al. investigated the effects of fullerenols with varying hydroxylation levels (C_60_(OH)*n*, where *n* = 0–40) on the aggregation of the NACore domain of alpha-synuclein, a key region involved in the pathology of PD. Using discrete molecular dynamics (DMD) simulations, they observed that amphiphilic fullerenols with 4–20 hydroxyl groups strongly inhibited NACore aggregation by forming hydrogen bonds with peptide backbones, which disrupted the formation of β-sheets [19]. Experimental validation using thioflavin-T (ThT) fluorescence assays, transmission electron microscopy (TEM), and Fourier transform infrared (FTIR) spectroscopy confirmed that C_60_(OH)_24_ effectively prevented amyloid fibril formation, whereas fully hydrophobic fullerenes and highly hydrophilic C_60_(OH)_40_ had little to no inhibitory effects [19]. The study further demonstrated that fullerenols reduce toxic β-barrel oligomer formation, an intermediate step in amyloid aggregation linked to neurotoxicity, supporting their potential as therapeutic inhibitors of neurodegenerative disease progression [19]. Importantly, in a study by Shi et al., visualization of the tissue distribution of fullerenols in zebrafish showed that fullerenol could also cross the blood-brain barrier [20], further supporting its promise as a neuroprotective drug candidate.

As mentioned above, in vitro and in vivo findings have confirmed the significant potential of fullerene and its derivatives in addressing the pathological hallmarks of AD. However, these studies have several limitations. For example, Kotelnicova et al. used C57BL/6 mice to study the potential effects of fullerene on cognition, extrapolating their conclusions to Alzheimer’s-specific pathology [8], whereas wild-type mice did not exhibit AD-like pathology. Andalib et al.’s study with fullerene was using the scopolamine model of AD [6], which also does not recapitulate histological hallmarks of AD, nor neuroinflammation, and oxidative stress [21]. Similarly, Aβ injection models do not mimic the histological features of AD and fail to replicate the chronic nature of AD, including its long-term neuroinflammatory responses.

Therefore, researchers are increasingly adopting more clinically relevant transgenic amyloid-beta animal models (e.g., APP/PS1, 3xTg-AD) to simulate AD’s intricate mechanisms, though these studies are scarce and have their serious limitations too [7,22]. For example, the study by Dai et al. used 3-month-old APP/PS1 transgenic mice to investigate the effects of fullerene C60 on AD-like pathology [7]. However, this study employed young mice and a very short treatment period of 10 days; it did not address the accumulation of amyloid, the key hallmark of AD, nor molecular changes that may underlie the reported behavioral effects. Additionally, Dai et al. used a complex method to prepare aqueous suspensions of fullerene and administered them intraperitoneally, which could pose challenges for clinical translation, particularly in terms of administration and reproducibility. Perovic et al. utilized the 5XFAD transgenic mouse model of Alzheimer’s disease and found that oral administration of a hydroxylated fullerene water complex (C_60_(OH)_24–45_, 0.15 g/L in distilled/tap water for three months) significantly decreased the amyloid-β plaque load in specific parts of the cerebral cortex [22]. However, this study did not include cognitive assessments, which may present challenges to future translational research. Thus, these studies underscore the need for more comprehensive research that incorporates both pathological and cognitive assessments in clinically relevant animal models to better understand the potential of fullerene-based treatments for Alzheimer’s disease. In this context, our study aimed to compare the potential of low-dose, long-term treatment with fullerene C_60_ and its water-soluble derivative fullerenol C_60_(OH)_24_, administered orally in equimolar concentrations (0.1 mg/kg/day and 0.15 mg/kg/day, respectively), to target key Alzheimer’s disease hallmarks in APP/PS1 mutant mice as potential preventive treatments for familial AD.

The choice of compounds, dosage, and treatment duration was based on previous efficacy data and our own in vitro findings, with particular attention to biosafety concerns associated with chronic toxicity effects at higher concentrations [23,24,25]. Therefore, we examined 12-month-old female APP/PS1 mice that were treated for 10 months with low doses of fullerene or fullerenol in a battery of tests for memory and emotionality and related these data to the molecular and histological AD-like changes in the brain. The latter was investigated using a refined approach to analyze amyloid plaques of various sizes: small (<100 μm^2^), medium (100–200 μm^2^), and large (>200 μm^2^). This approach is based on our previous observations of distinct functional roles related to behavioral abnormalities in APP/PS1 mice. Based on previous clinical and pre-clinical findings, we chose to study the expression of immune activation markers (*Il1β*, *Il6*, *Sqstm1*, and *Gdf15*) and cellular plasticity markers (*Sirt1*, *Tubβ3*, *Cldn5*, and *Bdnf*) in the hippocampus and prefrontal cortex of experimental groups of mice.

## 2. Materials and Methods

### 2.1. Synthesis of Fullerenol C_60_(OH)_24_ and Aβ42 Oligomers Preparation

A 300 mg portion of fullerene C_60_ (Sigma-Aldrich, Saint Louis, MO, USA) was saturated in 400 mL of benzene at room temperature for 24 h. The solution was filtered, and a solution of sodium hydroxide (20 g in 20 mL of water) and 2.5–3 mL of a 20% solution of tetrabutylammonium hydroxide was added to the filtrate while stirring (until the solution was decolorized). Benzene was removed from the reaction mixture under vacuum using a rotary evaporator. A total of 100 mL of water was added to the residue, and the resulting mixture was magnetically stirred for 15 h. The solution was filtered and the filtrate was evaporated to 50 mL using a rotary evaporator. After 300 mL of methanol was added, a dark brown flocculent precipitate was isolated, washed to neutral pH, and dried in a drying oven. The product obtained was fullerenol C_60_(OH)_24_ in 68% yield.

Oligomeric Aβ42 was prepared according to a protocol described previously [26,27]. Briefly, 1 mg of synthetic Aβ42 peptide (AnaSpec, Fremont, CA, USA) was dissolved in 200 μL of 100% 1,1,1,3,3,3-hexafluoro-2-propanol (HFIP) and aliquoted into 1.5 mL centrifuge tubes. HFIP was evaporated using an Eppendorf Concentrator Plus. The dried peptide was reconstituted in 2 μL of DMSO and diluted in ice-cold phenol-free Ham’s culture medium to a final concentration of 100 μM. The solution was sonicated for 60 s and incubated at 37 °C for 2 h.

### 2.2. Cell Culture

The CTX TNA2, bEnd.3, and BV2 cell lines were used in this study. CTX TNA2 cells are type 1 astrocytes (CRL-2006, ATCC, Manassas, VA, USA), bEnd.3 cells are mouse brain endothelial cells (CRL-2299, ATCC, Manassas, VA, USA), and BV2 cells are mouse microglial cell lines (ABC-TC212S, AcceGen, Fairfield, NJ, USA). All cell lines are widely used in neuroscience. Cells were cultured in DMEM (Gibco, Grand Island, NY, USA) supplemented with 10% fetal bovine serum (FBS, Gibco, Grand Island, NY, USA) and 1% penicillin/streptomycin (Gibco) and maintained in a humidified incubator at 37 °C with 5% CO_2_. To assess the cytotoxicity of C_60_(OH)_24_, cells were seeded in 96-well plates at a density of 2000 cells per well. The following day, fullerenol was added at concentrations ranging from 0.1 to 100 µg/mL and the cells were incubated for 48 h. To evaluate the influence of C_60_(OH)_24_ on ROS production, cells were treated with fullerenol for 24 h, followed by exposure to 5 μM Aβ42 for 60 min.

### 2.3. Cell Viability and ROS Production Assays

After 48 h, the number of viable cells was determined using a fluorescent cell counting kit (03285; Sigma-Aldrich, Saint Louis, MO, USA). Following exposure to C_60_(OH)_24_, the cells were rinsed with PBS, and 100 µL of PBS and 10 µL of the fluorescent working solution were added to each well. After a 60-minute incubation, the fluorescence intensity was measured at excitation and emission wavelengths of 485 nm and 535 nm, respectively, using a Hybrid Plate Reader Synergy H1 (Bio Tek, Winooski, VT, USA). The percentage of cell viability was calculated by normalizing to that of the control. ROS production in bEnd3, CTX TNA, and BV2 cells was measured using a cell-permeable dye (CM-H2DCFDA, C6827; Life Technologies, Carlsbad, CA, USA). The cells were pretreated with C_60_(OH)_24_ for 24 h. Then, the cells were incubated with CM-H2DCFDA (2.5 µM) for 60 min simultaneously with Aβ42 exposure. The fluorescence intensity of CM-H2DCFDA was measured using a microplate reader and was normalized to that of the control.

### 2.4. In Vivo Study with Chronic Dosing of APPswe/PS1dE9 Mice

#### 2.4.1. Animals and Study Flow

We used the APP/PS1 female mouse line (stock 034829, Jackson Laboratory, Bar Harbor, ME, USA) and age-matched wild-type (WT) littermates bred on a C57BL/6 background. Mice were housed 3–5 per cage under a reversed 12h light-dark cycle (lights on: 21:00) with free access to food and water under controlled laboratory conditions (22 ± 1 °C, 55% humidity). All experiments were performed according to the ethical guidelines of the U.S. Department of Health and Human Services (HHS) and the Registration of an Institutional Review Board (IRB) and were approved by the Ethics Committee of the Center for Life Sciences of Nazarbayev University (protocol No. 05-2023, 21 November 2023).

At the start of the experiment, the mice were two months old. For 10 months, APP/PS1 and WT groups were dosed with fullerene C_60_ (0.1 mg/kg/day) via self-made food pellets or with fullerenol C_60_(OH)_24_ (0.15 mg/kg/day) via tap water; one group of each genotype was vehicle-treated, i.e., received tap water. Randomization was conducted by body weight prior to the onset of treatment. Food pellets were prepared as described elsewhere [28]. The groups were as follows: WT vehicle n = 14, WT fullerene-treated n = 12, WT fullerenol-treated n = 13, APP/PS1 vehicle n = 9, APP/PS1 fullerene-treated n = 5, and APP/PS1 fullerenol-treated n = 6 at the age of 12 months; all mice were investigated in the conditioned taste aversion test, marble test, object recognition test, step-down avoidance, O-maze, dark-light box, and open field. Possible confounds in behavioral tests were systematically controlled by our laboratory. The day after the last behavioral test, mice were sacrificed (see below), and their brains were harvested for RT-PCR and histological analysis of amyloid plaque formation using Congo red staining (see below and Supplementary Figure S1). No criteria were set for including and excluding animals. Experimenters were blind to groups until the data analysis. In total, 59 mice were used; group sizes were calculated as described previously [28,29,30,31,32,33,34].

#### 2.4.2. Behavioral Assays

##### Conditioned Taste Aversion

In this paradigm, mice learn to associate a novel taste (2.5% sucrose solution) with nausea caused by lithium chloride injection, as described elsewhere [29]. During the training session, mice were deprived of water for 21 h and then allowed to drink a 2.5% sucrose solution for 30 min in a one-bottle paradigm. Thereafter, mice received an i.p. injection of lithium chloride (0.24 M) solution at a dose of 2% of body weight or PBS as a control. After injection, animals had access to sucrose solution for 1.5 h and were then water-deprived until the next day. Then, the mice were given a choice between tap water or 1% sucrose solution in a two-bottle paradigm for eight hours (recall test). The amount of liquid consumed was determined by weighing the bottles before and after each drinking session. Preference for sucrose solution, calculated as a percentage of the consumed sucrose versus the total amount of consumed liquid (sucrose preference = [amount of sucrose consumed/amount of sucrose and water consumed] × 100%), was taken as a measure of associative memory.

##### Marble Test

The tendency to displace small objects, such as small stones or food pellets, from a tube inside the cage is species-specific in mice and has been demonstrated to depend on intact hippocampal formation. Using a paper tube (internal diameter 4 cm, length 10 cm) filled with 20 food pellets and placed in the middle of a cage, the latency to displace the first food pellet and the number of pellets displaced in 15-min intervals were assessed as described elsewhere [30]. The total duration of the test was 90 min. During the first 20 min of the test, the number of displaced pellets was assessed every minute.

##### Object Recognition Test

The test was performed in a square box (45 × 45 × 45 cm) (Technosmart, Rome, Italy) and illuminated with white light (25 lx). On day 1, two identical objects were placed at opposite corners of the box, and the mice were allowed to explore them freely for 15 min. On day 2, one of the objects was replaced with a novel object. Latency to explore the novel object and duration of exploration were scored, and the discrimination index was calculated as follows: time to explore the novel object/total exploration time] × 100%, as described elsewhere [31].

##### Step-Down Avoidance

The step-down apparatus consisted of a transparent plastic cubicle (25 × 25 × 50 cm) with a stainless-steel grid floor (33 rods, 2 mm in diameter) (Technosmart, Rome, Italy), onto which a square wooden platform (7 × 7 × 1.5 cm) was placed. Mice were placed on a platform inside a transparent cylinder. On day 1, mice received a single electric shock (0.8 mA, 1 s) as soon as they left the platform and were immediately returned to their home cages. On day 2, mice were returned to the apparatus. After removal of the cylinder, the time until the animal left the platform with all four paws was scored as described elsewhere [32].

##### O-Maze

The maze consisted of a black circular path (runway width 5.5 cm, diameter 46 cm) (Technosmart, Rome, Italy) that was placed 20 cm above the floor, as described elsewhere [33]. Two opposing compartments were protected by walls (height, 10 cm). The illumination intensity was 5×. Mice were introduced into one of the two closed compartments. Latency and total number of exits to the open arms, risk assessment events, and total time spent in the open arms of the maze were scored for 5 min.

##### Dark-Light Box

The apparatus (OpenScience, Krasnogorsk, Russia) consisted of dark and illuminated (5 lx) compartments (both 20 × 20 × 25 cm). The mice were introduced into the dark compartment and allowed to move freely between the two chambers. Latency, total number of exits to the lit compartment, and total time spent in the compartment were scored for 5 min, as described elsewhere [30].

##### Open Field

The test was performed in a square box (45 × 45 × 45 cm) (Technosmart, Rome, Italy) illuminated with bright white light (600 lx). The animal was placed near the wall, and its movements were tracked for 15 min. The number of crossed sectors (16 total) as a measure of horizontal locomotor activity and the number of grooming events were calculated as described elsewhere [29,34].

#### 2.4.3. Euthanasia

Mice were terminally anesthetized and sacrificed by CO_2_ and isoflurane inhalation for subsequent material collection (RWD Life Science Co., Guangdong, China). Mice were first perfused with ice-cold saline via the left ventricle, halves of the brains were removed, and the hippocampi and prefrontal cortex were dissected and stored at −80 °C until use, as described elsewhere [35]. Brains were fixed in formalin solution for 24 h and used for histological analysis.

### 2.5. Brain Sectioning for Histological Assays

Brain tissue was washed three times for one hour and then dehydrated using graded ethanol solutions (75% for 1 h, 96% (I) for 5 min, 96% (II) for 5 min, 96% (III) for 5 min, 100% (I) for 5 min, and 100% (II) for 10 min). Each sample was incubated consecutively with 100% ethanol–chloroform (1:1) for 30 min, chloroform (I) for one h, and chloroform (II) overnight, and embedded in paraffin (three times, for one h each) at 60 °C, using a Leica EG1160 tissue embedding station (Leica Biosystems Inc., Nussloch, Germany). Paraffin sections were cut eight μm thick and mounted on polylysine-coated slides using a Leica RM 2265 microtome (Leica Biosystems Inc., Deer Park, IL, USA), as described previously [35].

### 2.6. Congo Red Staining, Plaque Microscopy, and Scoring of Amyloid Plaque Parameters

For tissue staining, the sections were deparaffinized in a xylene bath for 20 min, rehydrated using graded ethanol solutions (100% for 20 min, 95% for 5 min, and 50% for 5 min), washed three times with deionized water for 5 min, stained with 0.5% Congo red in 50% ethanol for 5 min, and differentiated with 0.2% KOH in 80% ethanol for 3 min. Slices were then embedded in the Epredia™ Immu-Mount™ water-based mounting medium (Thermo Fisher Scientific Inc., Kalamazoo, MI, USA). A total of 10 slices per animal were analyzed using confocal laser scanning microscopy LSM880 in the tile scan mode (Carl Zeiss, Oberkochen, Germany). To enable structure visualization, Congo red staining was combined with transmitted light photomultiplier (T-PMT) imaging [36]. The slice processing of morphological analysis of β-amyloid deposits was based on the QuPath 0.4.3 pixel classifier (Belfast, Northern Ireland, UK), an open-source software designed for digital pathology image analysis. Images were double-checked to remove artifacts and folds [37]. The analysis of the fluorescent image was carried out in one channel of red color, compared to the T-PMT-negative dots. The selection of areas with brightness above the threshold border was performed pixel by pixel. The total number of detected plaques was obtained and the area of each plaque in each ROI was calculated using the algorithm. The dimensions of these regions were determined and validated using a histology atlas [38]. Bioimage analysis revealed amyloid plaques of various size ranges: small, <100 μm^2^, medium, 100–200 μm^2^, and large >200 μm^2^. The density of each plaque type was calculated per mm^2^ in each of the examined brain regions. The total area, number of plaques, and maximum single plaque size per square were also recorded. For each animal, we obtained minimal (min), 1st quartile (Q1), 2nd quartile (Q2), 3rd quartile (Q3), and maximal (max) values. This resulted in 5 statistical samples: min, Q1, Q2, Q3, and max. To summarize these data, we calculated the mean value of each sample, which represents the average minimum, Q1, Q2, and Q3, and maximum plaque sizes across the group.

### 2.7. Immunohistochemical Analysis of Astrocyte Activation

Immunostaining procedure was performed overnight in a humidified chamber at +4 °C as described previously [39]. Briefly, the brain’s 8 μm thick sections were stained with primary anti-GFAP (Glial fibrillary acidic protein) antibody (ab7260, Recombinant Full-Length Protein corresponding to Human/Mouse GFAP, Rabbit polyclonal (Abcam, Waltham, MA, USA, diluted 1:1000) and secondary Goat anti-Rabbit IgG (H + L) antibodies (A11011, Alexa Fluor™ 568, Invitogen™, Thermo Fisher Scientific Inc., Carlsbad, CA, USA, diluted 1:1000). Nuclei were counterstained with DAPI (62248, Thermo Fisher Scientific Inc., Carlsbad, CA, USA, diluted 1:1000) and embedded in the Epredia™ Immu-Mount™ water-based mounting medium (Thermo Fisher Scientific Inc., Kalamazoo, MI, USA).

### 2.8. RNA Extraction, cDNA Synthesis, and Real-Time Polymerase Chain Reaction

Total RNA of the prefrontal cortex and hippocampus tissue samples was isolated using QIAzol^®^ Lysis Reagent (QIAGEN Sciences Inc., Germantown, MD, USA). Tissue was placed in 1 mL of QIAzol, homogenized using a TissueRuptor (QIAGEN Sciences Inc., Germantown, MD, USA) with two 30 s cycles of the homogenizer at half speed, and then placed on ice for one minute after every homogenization. Homogenized samples were centrifuged for 15 min at 12,000× *g* and 4 °C to remove any remaining cell debris. Chloroform was then added to the homogenized sample and centrifuged for 15 min at 12,000× *g* at 4 °C, and the aqueous phase was carefully removed. RNA precipitation was performed with the addition of ethanol, and the subsequent RNA pellet was washed and cleaned using the RNeasy Mini Kit (QIAGEN Sciences Inc., Germantown, MD, USA). The RNA concentration was measured using a NanoDrop spectrophotometer (Thermo Fisher Scientific, Waltham, MA, USA).

The total RNA (1 μg) was converted to cDNA. First-strand cDNA synthesis was performed using random primers and a QuantiTect Reverse Transcription Kit (QIAGEN Sciences Inc., Germantown, MD, USA). The reaction was performed in an Eppendorf Mastercycler^®^ Gradient using the following temperature program: 68 °C for 5 min, 42 °C for 60 min, and 70 °C for 10 min. To identify the expression levels of the target genes, we designed gene-specific primers. The housekeeping glyceraldehyde 3-phosphate dehydrogenase (*Gapdh)* gene was used as a reference. The sequences of the designed primers are listed in Table S1 (Supplementary File). The expression levels of immune activation and cellular plasticity markers were determined using real-time polymerase chain reaction (RT-PCR). RT-PCR was performed using the SYBR Green Master Mix (Applied Biosystems SYBR Green Universal Master Mix, Foster City, CA, USA). RT-PCR was performed in a 10 μL reaction volume containing SYBR Green master mix (5 µL), RNase-free water (3 μL), specific forward and reverse primers used at a concentration of 20 pmol/µL (1 μL), and cDNA (1 μL). The initial denaturation step for RT-PCR was performed at 95 °C for 5 min followed by 40 cycles of denaturation at 95 °C for 20 s, annealing at 60 °C for 30 s, and extension at 68 °C for 30 s. All primers were purchased from Sigma-Aldrich (USA). All samples were run in triplicate. Reactions were performed using an ABI Prism 7900 HT SDS instrument (Applied Biosystems, Foster City, CA, USA).

### 2.9. Statistical Analysis

For in vitro studies, data from at least three independent experiments were averaged, normalized to the control, and presented as the mean ± SD. One-way analysis of variance (ANOVA) was used to investigate the effects of fullerenol on cell viability and ROS formation. When one-way ANOVA was significant, a post hoc test using Tukey’s and Dunnett’s pairwise comparison of means was used to reveal differences between various groups.

The in vivo data were analyzed using a statistical software package (GraphPad PRISM 8.0.2, San Diego, CA, USA). The Shapiro-Wilk test for normality was used. Two-way ANOVA followed by a post hoc Tukey’s test was performed for the comparison of quartiles. A one-sample *t*-test was used to reveal differences from the chance level of discrimination index in study groups. No datapoints were excluded from analysis. The significance level was set at 95% (*p* < 0.05). Data are presented as the mean ±SEM; group sizes are indicated in the Materials and Methods and Figure legends.

## 3. Results

### 3.1. Effects of Fullerenol on Cell Viability and ROS Production

**Figure 1:**
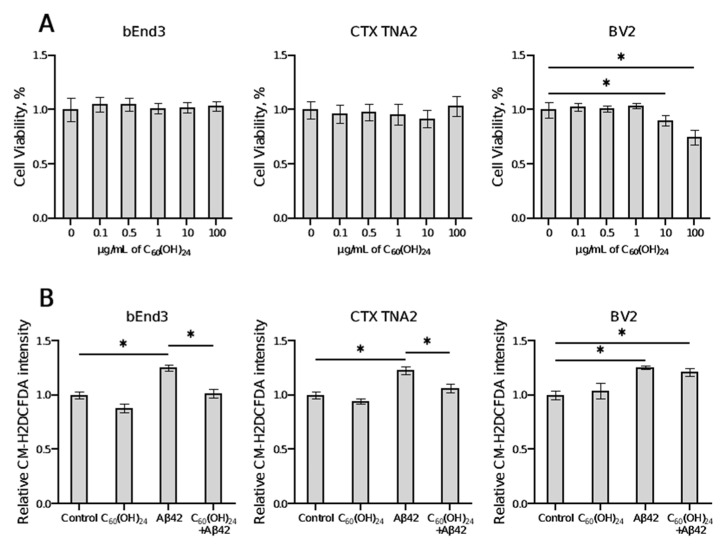
Impact of fullerenol on cellular viability and reactive oxygen species (ROS) production in vitro. The percentage of viability in bEnd3, CTX TNA2, and BV2 cells is depicted in (**A**), while the level of ROS in bEnd3, CTX TNA2, and BV2 cells is shown in (**B**). Statistical significance is indicated by * *p* ≤ 0.05, in comparison to the control group and the group exposed solely to Aβ42.

To assess the effect of fullerenol on cell viability, we incubated brain endothelial cells (bEnd3), astrocytes (CTX TNA2), and microglial cells (BV2) with fullerenol for 48 h in concentrations ranging from 0.1 μg/mL to 100 μg/mL. The results of the cell viability assay demonstrated that 48 h of fullerenol treatment had no cytotoxic effects at any of the studied concentrations in endothelial cells and astrocytes. For bEnd3 and CTX TNA2, the percentage of viable cells was not significantly different between the experimental and control groups (Figure 1A). In microglial cells, fullerenol at lower concentrations (0.1–1 μg/mL) did not significantly affect cell viability compared to the control. However, at higher concentrations of 10 μg/mL and 100 μg/mL, fullerenol reduced cell viability by approximately 10% (*p* = 0.0363, Dunnett’s test) and 26% (*p* < 0.0001, Dunnett’s test), respectively (Figure 1A).

To assess the effect of fullerenol on Aβ42-induced ROS production, cells were pretreated with C_60_(OH)_24_ and then incubated with Aβ42 (Figure 1B). In agreement with previous studies, Aβ42 exposure increased ROS production in endothelial cells (~25%, *p* < 0.0001, Tukey’s test), astrocytes (~22%, *p* < 0.0001, Tukey’s test), and microglial cells (~25%, *p* < 0.0001, Tukey’s test). Pretreatment with fullerenol significantly reduced Aβ42-induced ROS levels, normalizing them to near control levels in bEnd3 (*p* < 0.0001, Tukey’s test, Figure 1B) and CTX TNA2 cells (*p* < 0.0001, Tukey’s test, Figure 1B). However, in BV2 cells, fullerenol pretreatment had no significant effect on Aβ42-induced ROS production; the ROS level in this group remained elevated by approximately 21% compared to control (*p* < 0.0001, Tukey’s test, Figure 1B). Fullerenol treatment alone did not increase ROS levels compared to the control.

### 3.2. Differential Effects of Fullerene and Fullerenol on Hippocampus-Related Cognitive Performance in APP/PS1 Mice

**Figure 2:**
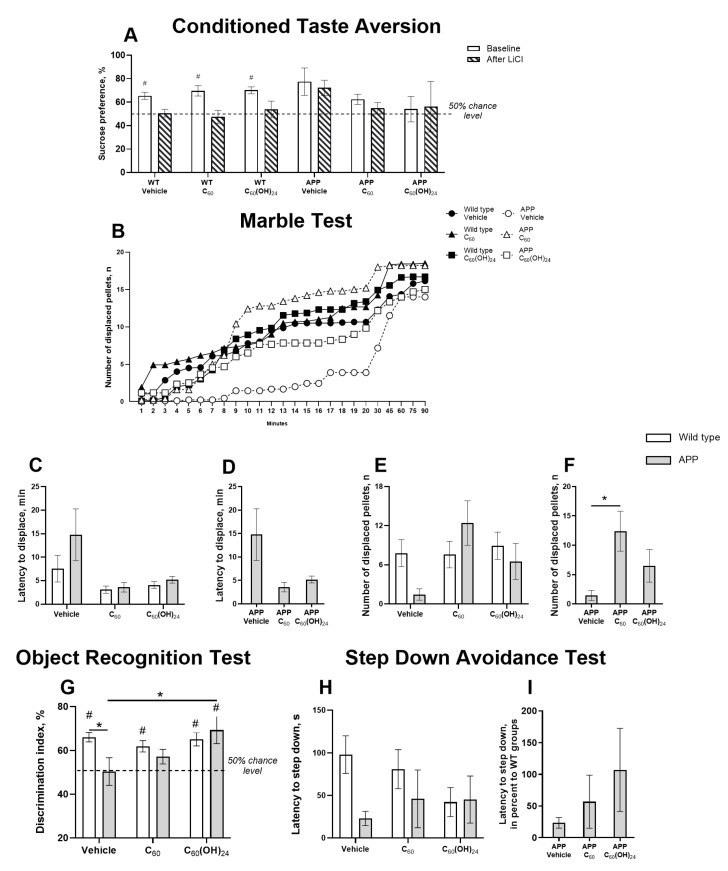
Effects of the APP/PS1 mutation and treatment with fullerene compounds on associative memory and hippocampal-dependent behavior. (**A**) After injection of LiCl, the sucrose preference in the conditioned taste aversion test was not significantly different from the chance level in non-treated, fullerene-, and fullerenol-treated WT groups, suggesting their avoidance of sucrose taste, but not in vehicle-treated mutants that showed elevated sucrose preference. (**B**) Number of displaced pellets during the marble test. (**C**) No significant differences between groups were demonstrated in the latency to displace the first pellet. No significant group differences in this parameter were found between mutant mice in (**D**) absolute values. (**E**) No significant differences between the groups were found in the number of displaced pellets after the first 10 min of the test. This measure was significantly higher in the fullerene-treated group than in the non-treated mice in (**F**) absolute values. (**G**) In the object recognition test, the discrimination index was significantly lower in non-treated mutant mice than in untreated WT and fullerenol-treated mutants. The discrimination index significantly differed from the chance level in non-treated, fullerene-, and fullerenol-treated WT mice, as well as in fullerenol-treated mutants. In the latency to descend from the platform in the step-down avoidance test, no significant differences were observed between (**H**) all experimental groups and (**I**) mutants only. WT—wild types. Two-way and one-way ANOVA and one-sample *t*-test. * *p* < 0.05, post hoc Tukey’s test. # *p* < 0.05, one-sample *t*-test.

Repeated-measures ANOVA revealed a significant effect of training in the conditioned taste aversion test (F = 6.028, *p* = 0.0197, two-way ANOVA; Figure 2A), but not the group factor or their interaction (F = 1.49, *p* = 0.229 and F = 0.66, *p* = 0.65, respectively). Although no significant group differences were found, a strong trend toward reduced sucrose preference was noted in WT mice treated with C_60_ (*p* = 0.0579, Tukey’s test). Baseline sucrose preference was significantly above the chance level in all WT groups (WT Vehicle: *p* = 0.0017, WT-C_60_: *p* = 0.007; WT-C_60_(OH)_24_: *p* = 0.0003, one-sample *t*-test, respectively; Figure 2A), but this was abolished after LiCl injection (WT Vehicle: *p* = 0.8765, WT-C_60_: *p* = 0.6557; WT-C_60_(OH)_24_: *p* = 0.62, one-sample *t*-test, respectively; Figure 2A). In APP/PS1 mice, no significant treatment effects were observed, suggesting a limited impact of administration of C_60_ or C_60_(OH)_24_ on conditioned taste aversion learning (Figure S2A,B).

In the marble test, significant genotype effects were found in the number of displaced pellets (F = 28.38, *p* < 0.0001, two-way ANOVA; Figure 2B), with a decreased number of displacements in the APP Vehicle group from 12 to 17 min in comparison to the WT Vehicle, C_60_, and C_60_(OH)_24_ groups (*p* < 0.05, Tukey’s test; Figure 2B), and from 10 to 20 min (*p* < 0.05, Tukey’s test; Figure 2B) compared to APP C_60_. Significant treatment effects but no genotype effect or their interaction were found in the latency to displace the first pellet (F = 4.48, *p* = 0.0161, F = 1.48, *p* = 0.229, and F = 0.88, *p* = 0.418, respectively; two-way ANOVA; Figure 2C). This measure did not differ significantly between mutants in absolute values (F = 1.862, *p* = 0.187, one-way ANOVA; Figure 2D). The genotype effect was not significant for the number of displaced pellets after 10 min (F = 0.45, *p* = 0.5); strong trends for the treatment effect and genotype × treatment interaction were observed (F = 2.71, *p* = 0.075 and F = 2.8, *p* = 0.069, respectively, two-way ANOVA; Figure 2E). Significant group differences were found for this measure between mutants (F = 6.4, *p* = 0.0084, one-way ANOVA); in fullerene-treated mutants, it was significantly higher than that in non-treated mutants (*p* = 0.0065, Tukey’s test; Figure 2F). Significant group differences were also observed for this measure in percentage to respective WT groups (F = 7.31, *p* = 0.0051, one-way ANOVA). These results suggest partial rescue of hippocampus-dependent performance by fullerene administration.

Two-way ANOVA revealed a significant genotype × treatment interaction for the discrimination index in the object recognition test (F = 3.28, *p* = 0.0454) and a strong trend for the treatment effect (F = 2.92, *p* = 0.0627) but no significant genotype effect (F = 2.61, *p* = 0.112). The discrimination index was significantly lower in non-treated mutants than in non-treated WT mice (*p* = 0.04, Tukey’s test), but fullerenol-treated mutants showed a significant increase compared with the non-treated group (*p* = 0.042; Figure 2G). A one-sample *t*-test revealed a significant difference from the chance level of discrimination index in non-treated, fullerene-, and fullerenol-treated WT groups (*p* < 0.0001, *p* = 0.008, and *p* = 0.003, respectively), as well as in fullerenol-treated mutants (*p* = 0.03); a strong trend for such a difference was also observed in fullerene-treated mutants (*p* = 0.098; Figure 2G). This indicates a beneficial effect of both treatments, particularly fullerenol, on object recognition memory.

In the step-down avoidance test, no significant treatment effect (F = 0.41, *p* = 0.66, two-way ANOVA) or genotype × treatment interaction (F = 1.55, *p* = 0.22) was found for latency to step down, but a strong trend for genotype effect was observed (F = 3.5, *p* = 0.066; Figure 2H). Between mutants, no significant differences were found in this measure in percentage compared to the respective WT groups (F = 1.26, *p* = 0.31, one-way ANOVA; Figure 2I).

### 3.3. Chronic Fullerene and Fullerenol Treatment Partially Improves Emotionality in APP/PS1 Mice Without Significantly Affecting Locomotion

**Figure 3:**
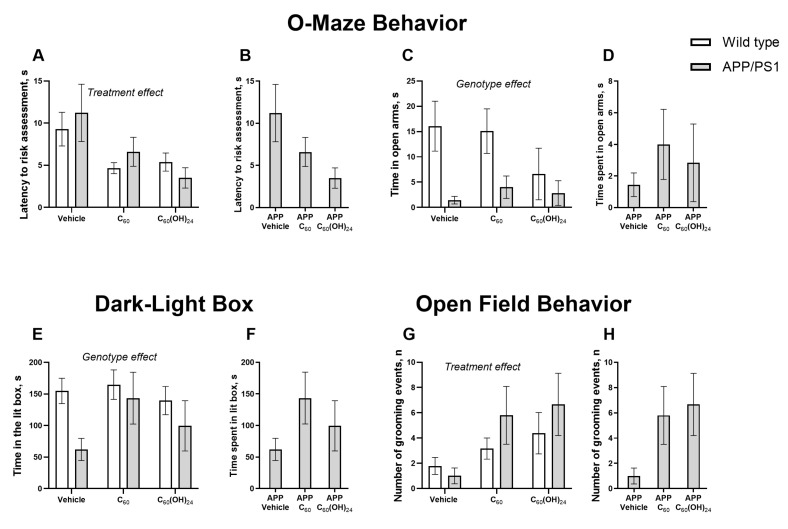
Effects of treatment with fullerene compounds on the emotionality of APP/PS1 mice. In O-maze, no significant differences between groups were found in (**A**) latency-to-risk assessment and this measure between mutants in (**B**) absolute values; in (**C**) time spent in open arms and this measure between mutants in (**D**) absolute values. In the dark-light box, no significant differences between groups were found in (**E**) time spent in the lit box, and this measure between mutants in (**F**) absolute values. In the open field, no significant differences between groups were found in the (**G**) number of grooming events and this measure between mutants in (**H**) absolute values. Two-way and one-way ANOVA variance.

In the O-maze test, significant treatment effects were found in the latency-to-risk assessment (F = 5.08, *p* = 0.0069, two-way ANOVA; Figure 3A), but no significant effects of genotype or genotype × treatment interaction were observed (F = 0.16, *p* = 0.69, and F = 0.57, *p* = 0.56, respectively). No significant differences in this parameter were found between mutants in absolute values (F = 2.03, *p* = 0.163, one-way ANOVA; Figure 3B). No significant effects of genotype, treatment, or their interaction were demonstrated for the number of exits to the lit box (F = 0.34, *p* = 0.56; F = 0.29, *p* = 0.75; and F = 2.26, *p* = 0.12, respectively; two-way ANOVA; see Supplementary Figure S3A). No significant differences in this measure were found between mutants in absolute values (F = 1.12, *p* = 0.345, one-way ANOVA; see Supplementary Figure S3B) and in percentage to respective WT groups (F = 2.33, *p* = 0.127; see Supplementary Figure S3C). In the time spent in open arms, a genotype effect was found in the time spent in the open arms (F = 5.72, *p* = 0.0203, two-way ANOVA), but no treatment effect or genotype × treatment effects were observed (F = 0.51, *p* = 0.604 and F = 0.65, *p* = 0.525, respectively; Figure 3C). No significant differences in this parameter were found between mutants in absolute values (F = 0.58, *p* = 0.56, one-way ANOVA; Figure 3D).

In the dark box, a significant genotype effect was revealed for time spent in the lit box (F = 5.44, *p* = 0.0235, two-way ANOVA), but no effects of treatment or genotype × treatment interaction were observed (F = 1.49, *p* = 0.234 and F = 1.02, *p* = 0.366, respectively; Figure 3E). No significant differences in this measure were found between mutants in absolute values (F = 1.77, *p* = 0.2, one-way ANOVA; Figure 3F).

In the open field, there was a significant treatment effect (F = 4.29, *p* = 0.019), but not a genotype effect or genotype x treatment interaction (F = 1.25, *p* = 0.268 and F = 0.8, *p* = 0.45, respectively, two-way ANOVA; Figure 3G) in the number of grooming events. No significant differences in this measure were found between mutants in absolute values (F = 2.58, *p* = 0.111, one-way ANOVA; Figure 3H). Two-way ANOVA did not reveal any significant effects of genotype, treatment, or their interaction on the number of crossed sectors (F = 0.18, *p* = 0.97; F = 0.3, *p* = 0.74; and F = 0.31, *p* = 0.73, respectively; see Supplementary Figure S3D–F).

### 3.4. Distribution of Amyloid Plaques of Various Sizes Across the Cortex, Hippocampus, and Thalamus Under the Influence of Fullerenol

**Figure 4:**
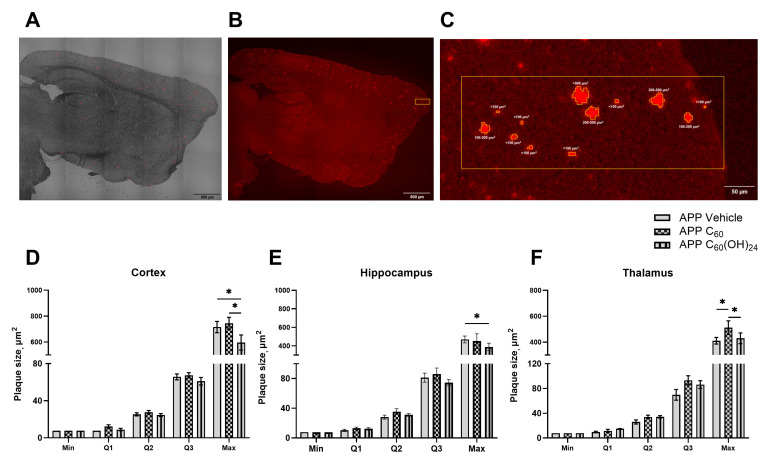
Chronic administration of fullerenol, as opposed to fullerene, results in a reduction in amyloid plaque quartiles in the brain. For each animal, minimal (min), 1st quartile (Q1), 2nd quartile (Q2), 3rd quartile (Q3), and maximal (max) values were defined. (**A**) Representative brain sections stained with Congo red in APP/PS1 mice. Tile scan of Congo red staining for amyloid formation in the brain, combined with T-PMT signaling at ×10 magnification, scale bar—800 µm. (**B**) Tile scan of Congo red staining for amyloid plaque formation in the brains of APP/PS1 mice, x10 magnification, scale bar—800 µm. (**C**) Congo red-stained amyloid plaques categorized by size: very small (<100 μm^2^), small (100–200 μm^2^), medium (200–500 μm^2^), and large (>500 μm^2^), scale bar—50 µm. Significant differences in maximum plaque size were observed (**D**) between APP/PS1 Vehicle and APP/PS1 C_60_(OH)_24_, and between APP/PS1 C_60_ and APP/PS1 C_60_(OH)_24_ in the cortex, (**E**) between APP/PS1 Vehicle and APP/PS1 C_60_(OH)_24_ in the hippocampus, and (**F**) between APP/PS1 C_60_ and APP/PS1 C_60_(OH)_24_, as well as between APP/PS1 Vehicle and APP/PS1 C_60_ groups in the thalamus. Two-way ANOVA followed by post hoc Tukey’s tests, * *p* < 0.05.

Congo red staining revealed that in all examined brain regions of APP/PS1 mice, plaque inclusions of varying sizes were extensively distributed throughout the brain parenchyma (Figure 4A,B). The proportion of plaques was consistent across all brain structures. On average, approximately 80% of all plaques measured less than 100 µm^2^ in area, around 10% ranged between 100 and 200 µm^2^, and approximately 10% were large plaques exceeding 200 µm^2^. The administration of C_60_ and C_60_(OH)_24_ did not significantly affect the percentage distribution of plaque sizes (see Supplementary Figure S4). A two-way ANOVA indicated a strong trend for treatment effect on percentile values in the cortex (F = 2.765, *p* = 0.0699, two-way ANOVA, Figure 4D) and thalamus (F = 2.378, *p* = 0.0757, two-way ANOVA, Figure 4F), but not in the hippocampus (F = 0.8192, *p* = 0.4450, two-way ANOVA, Figure 4E). A significant interaction was observed in the cortex (F = 2.345, *p* = 0.027, two-way ANOVA, Figure 4D), but not in the hippocampus (F= 0.675, *p* = 0.7118, two-way ANOVA, Figure 4E) or thalamus (F = 1.524, *p* = 0.1648, two-way ANOVA, Figure 4F). Post hoc analysis revealed a significant decrease in the maximum plaque size in the cortex of the C_60_(OH)_24_-treated APP/PS1 group compared to the vehicle APP/PS1 group (*p* = 0.0008, Tukey’s test, Figure 3D) and the C_60_-treated APP/PS1 group (*p* < 0.0001, Tukey’s test, Figure 4D). A significant increase in the maximum plaque size in the thalamus was found in the C_60_-treated APP/PS1 mice compared to the vehicle APP/PS1 group (*p* = 0.0007, Tukey’s test, Figure 3F) and the C_60_(OH)_24_-treated APP/PS1 animals (*p* = 0.0069, Tukey’s test, Figure 4F).

### 3.5. Beneficial Effects of Treatment with Fullerenol but Not Fullerene on Plaque Deposition in the Brain

**Figure 5:**
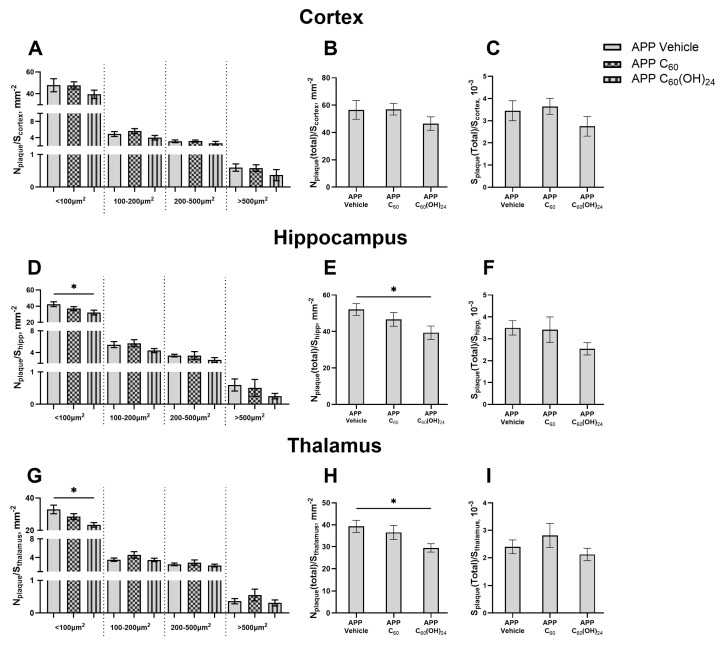
Amyloid plaque density of various sizes in brain regions of APP/PS1 mice. Statistical analyses using one-way ANOVA followed by post hoc Dunnett’s tests or the Kruskal-Wallis test followed by post hoc Dunn’s test revealed no significant differences in (**A**) the density of plaques of different sizes, (**B**) the total density of plaques, and (**C**) the total area of plaques, normalized to cortex area, among the APP/PS1 Vehicle, APP/PS1 C_60_, and APP/PS1 C_60_(OH)_24_ groups in the cortex. In contrast, one-way ANOVA followed by post hoc Dunnett’s test indicated a significant reduction in (**D**) the density of the smallest (<100 μm^2^) plaques and (**E**) the total density of plaques, but not in (**F**) the total area of plaques, within the APP/PS1 C_60_(OH)_24_ group in the hippocampus (* *p* < 0.05). Similarly, one-way ANOVA followed by post hoc Dunnett’s tests demonstrated a significant decrease in (**G**) the density of the smallest (<100 μm^2^) plaques and (**H**) the total density of plaques, but not in (**I**) the total area of plaques, in the APP/PS1 C_60_(OH)_24_ group in the thalamus. * *p* < 0.05.

One-way ANOVA indicated no significant treatment effects on the density of cortical plaques of sizes <100, 100–200, and 200–500 μm^2^ (F = 1.057, *p* = 0.3738; F = 1.785, *p* = 0.2039; and F = 0.6320, *p* = 0.5461, respectively, one-way ANOVA, Figure 5A), as well as >500 μm^2^ (*p* = 0.2031, Kruskal-Wallis test, Figure 4A). The treatment effect was also not significant for total plaque density and total plaque area, normalized to zone area, in the cortex (F = 1.134, *p* = 0.3496 and F = 1.213, *p* = 0.3266, respectively, one-way ANOVA, Figure 5B,C).

There was a notable trend indicating a treatment effect on the density of the smallest plaques (<100 μm^2^) in the hippocampus (F = 3.442, *p* = 0.0608, one-way ANOVA, Figure 5D) and a significant treatment effect in the thalamus (F = 5.736, *p* = 0.0151, one-way ANOVA, Figure 5G). Post hoc analysis revealed a significant reduction in this measure in the C_60_(OH)_24_-treated APP/PS1 group in both the hippocampus and thalamus (*p* = 0.0369 and *p* = 0.0084, respectively, Dunnett’s test, Figure 5D,G), whereas no significant changes were observed in the C_60_-treated group (*p* = 0.3324 and *p* = 0.2614, respectively, Dunnett’s test, Figure 5D,G). There were no significant alterations in the density of plaques measuring 100–200, 200–500, and >500 μm^2^ in the hippocampus (F = 1.931, *p* = 0.1817; F = 1.010, *p* = 0.3891; and F = 1.008, *p* = 0.3839, respectively, one-way ANOVA, Figure 5D) and thalamus (F = 1.494, *p* = 0.2582; F = 0.6258, *p* = 0.5491; and F = 1.114, *p* = 0.3558, respectively, one-way ANOVA, Figure 5G). One-way ANOVA indicated a strong trend for a treatment effect on total plaque density, but not on the total area of plaques normalized to zone area, in the hippocampus (F = 3.430, *p* = 0.0613 and F = 1.874, *p* = 0.19, respectively, one-way ANOVA, Figure 5E,F). Post hoc testing demonstrated a significant decrease in total plaque density in the hippocampus for the C_60_(OH)_24_-treated APP/PS1 group (*p* = 0.0376, Dunnett’s test, Figure 5E), but not for the C_60_-treated group (*p* = 0.4828, Dunnett’s test, Figure 5E). A significant treatment effect was observed in the total plaque density, but not in the total area of plaques normalized to zone area, in the thalamus (F = 3.989, *p* = 0.0425 and F = 1.271, *p* = 0.3110, respectively, one-way ANOVA, Figure 5H,I). Post hoc analysis showed a significant reduction in total plaque density in the thalamus for the C_60_(OH)_24_-treated APP/PS1 group (*p* = 0.0289, Dunnett’s test, Figure 5H), but not for the C_60_-treated group (*p* = 0.6892, Dunnett’s test, Figure 5H).

### 3.6. The Impact of the Treatment on the Number of GFAP-Positive Cells in the Brain

**Figure 6:**
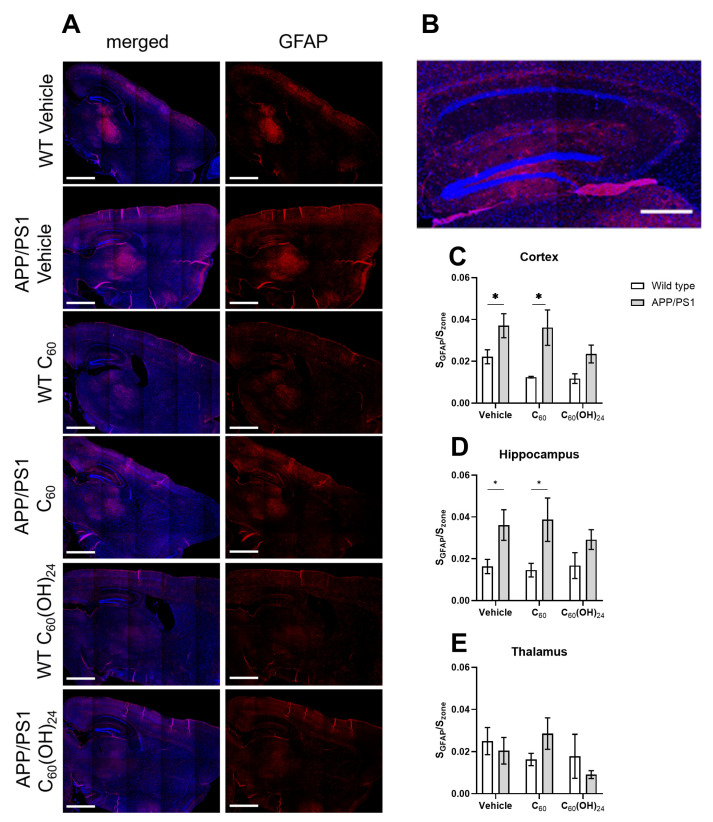
Immunohistochemical analysis of astrocyte activation in brain regions of WT and APP/PS1 mice. (**A**) Representative images of mouse brain stained with anti-GFAP staining (red signal), as a marker of astrocytes, merged with DAPI nuclei staining (blue signal). The scale bar = 1000 um. (**B**) Representative image of mouse hippocampus stained with anti-GFAP staining (red signal), as a marker of astrocytes, merged with DAPI nuclei staining (blue signal). The scale bar = 500 um. (**C**) In the cortex, the density of GFAP-positive cells was markedly higher in APP/PS1 mice than in WT mice (* *p* < 0.05, post hoc Holm–Šídák’s test). The APP/PS1 group treated with C60 had increased density of GFAP cells (* *p* < 0.05). (**D**) In the hippocampus, the density of GFAP-positive cells was significantly higher in APP/PS1 mice than in WT mice (* *p* < 0.05), and significantly increased in APP/PS1-C60 mice compared to treated WT-C60 mice (* *p* < 0.05). The APP/PS1 C60(OH)24 group displayed no significant changes in the density of GFAP-positive cells in the cortex and hippocampus, suggesting a partial attenuation of astrogliosis in these animals. (**E**) In the thalamus, the number of GFAP-positive cells remained unchanged in all groups of APP/PS1 mice regardless of the treatment.

The representative images of the brain sections stained with anti-GFAP are shown in Figure 6A,B, and the corresponding quantitative analysis is presented in Figure 6C–E. The number of GFAP-positive cells, in the APP/PS1 mice model, an indicator of astrocyte activation, was significantly higher in the cortex and hippocampal regions compared to control WT mice (*p* = 0.0306 and *p* = 0.0271, respectively, one-way ANOVA with post hoc Holm–Šídák’s test, Figure 6C,D), but not in the thalamus (Figure 6E). This increase was also significantly upregulated in the C60-treated APP/PS1 mice group compared to the C60-treated WT controls (*p* = 0.0034 and *p* = 0.0172, respectively, one-way ANOVA with post hoc Holm–Šídák’s test, Figure 6C,D). In contrast, C_60_(OH)_24_-treated APP/PS1 mice showed a trend towards downregulated GFAP activation compared to C_60_(OH)_24_-treated WT mice. The results of this study suggest that astrocyte activation, as indicated by increased GFAP expression, is elevated in APP/PS1 mice but is reduced following C_60_(OH)_24_ treatment.

### 3.7. Region-Specific Modulation of Inflammatory and Plasticity-Related Gene Expression by Fullerene and Fullerenol in APP/PS1 Mice

**Figure 7:**
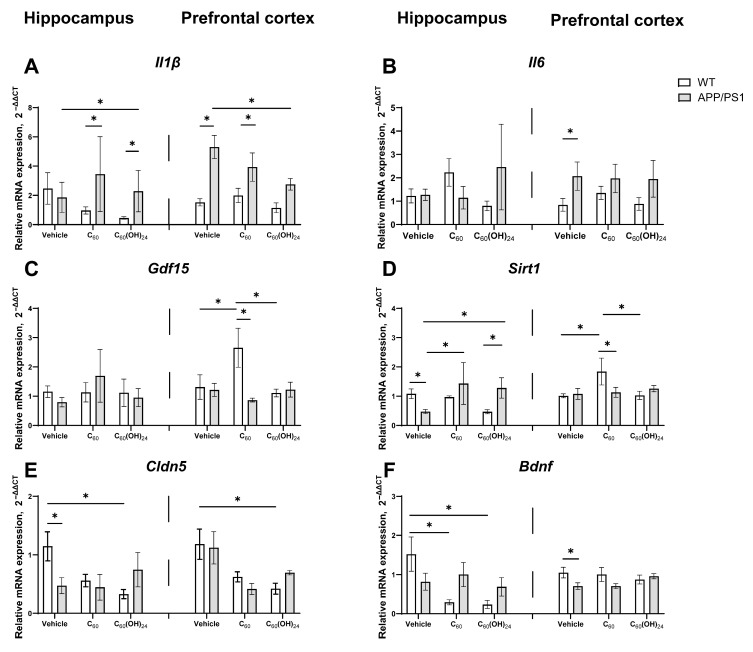
Relative mRNA expression of selected markers related to inflammation, aging, and plasticity in the prefrontal cortex and hippocampus of APP/PS1 and WT mice. (**A**–**C**) Expression profile of inflammation-related genes in the prefrontal cortex (*Il1β*, *Il6*, *Gdf15*) in the hippocampus and prefrontal cortex. (**D**–**F**) Expression profile of neuronal dysfunction-related genes (*Sirt1*, *Cldn5*, *Bdnf*) in the hippocampus and prefrontal cortex. WT—white bar; APP/PS1—grey bar; * *p* < 0.05.

Figure 7 presents the effects of fullerene and fullerenol on the gene expression of immune activation markers *(Il1β, Il6*, and *Gdf15*) and cellular plasticity markers (*Sirt1, Cldn5*, and *Bdnf*) in the prefrontal cortex and hippocampus of APP/PS1 and WT mice. In the hippocampus, a significant genotype effect on *Il1β* expression was identified (F = 17.45, *p* < 0.001, two-way ANOVA). In transgenic mice, both fullerene and fullerenol led to an increase in *Il1β* expression compared to WT-treated animals (*p* = 0.0120, *p* < 0.05, and *p* = 0.0020, *p* < 0.01, respectively; Tukey’s test; Figure 7A). Furthermore, fullerenol-treated mice had elevated *Il1β* expression in the APP/PS1-treated group relative to the APP/PS1 vehicle group (*p* = 0.0454, *p* < 0.05, Tukey’s test; Figure 7A). In the prefrontal cortex, a significant genotype effect was also observed (F = 31.46, *p* < 0.001, two-way ANOVA). *Il1β* expression was significantly higher in APP/PS1 mice compared to WT mice in the vehicle-treated group (*p* = 0.0004, *p* < 0.001, Tukey’s test; Figure 7A) and in the fullerene-treated group (*p* = 0.0185, *p* < 0.05, Tukey’s test; Figure 7A). However, no significant difference in *Il1β* levels was observed in APP/PS1 mice treated with fullerenol compared to WT mice. Additionally, *Il1β* levels were reduced in the fullerenol-treated APP/PS1 group compared to the Vehicle group (*p* = 0.0460, *p* < 0.05, Tukey’s test; Figure 7A).

*Il6* expression did not demonstrate significant differences between groups in the hippocampus (Figure 7B). In contrast, a significant genotype effect was identified in the prefrontal cortex (F = 11.51, *p* < 0.01, two-way ANOVA), although no significant treatment effect or genotype × treatment interaction was observed (F = 0.03058, *p* = 0.9699, and F = 0.08948, *p* = 0.9153, respectively; Figure 7B). Similarly, *Gdf15* expression did not exhibit significant differences between groups in the hippocampus (Figure 7C). However, in the prefrontal cortex, both a significant genotype effect (F = 4.473, *p* < 0.05, two-way ANOVA) and a significant genotype × treatment interaction (F = 4.196, *p* < 0.05, two-way ANOVA) were observed. *Gdf15* levels were significantly elevated in wild-type mice treated with C_60_ compared to APP/PS1-vehicle-treated mice (*p* = 0.0024, *p* < 0.01, Tukey’s test; Figure 7C), as well as compared to the WT control and C_60_(OH)_24_ groups (*p* = 0.033, *p* < 0.05, and *p* = 0.0156, *p* < 0.05, respectively; Tukey’s test; Figure 7C).

In the analysis of *Sirt1* expression levels in the hippocampus, a significant interaction effect between genotype and treatment was identified (F = 6.364, *p* = 0.0058, two-way ANOVA). Subsequent post hoc analysis indicated that *Sirt1* expression was markedly elevated in the wild-type vehicle group compared to untreated transgenic mice (*p* = 0.0288, Tukey’s test; Figure 7D). Conversely, treatment with C_60_ and C_60_(OH)_24_ resulted in a significant increase in *Sirt1* expression in APP/PS1 mice relative to the control group (*p* = 0.0288, and *p* = 0.0454, respectively; Tukey’s test; Figure 7D), suggesting a potential restorative effect of fullerenes on *Sirt1* levels in the Alzheimer’s disease model. Additionally, Sirt1 expression in fullerenol-treated transgenic mice exceeded that in wild-type mice (*p* = 0.0201, Tukey’s test; Figure 7D). In the prefrontal cortex, two-way ANOVA revealed no significant effects of genotype, treatment, or their interaction on Sirt1 expression. However, post hoc analysis demonstrated that *Sirt1* expression was significantly higher in the fullerene-treated wild-type group compared to the APP/PS1 group (*p* = 0.0218, Tukey’s test; Figure 7D). Furthermore, *Sirt1* expression was significantly elevated in the wild-type C_60_-treated group compared to both the vehicle and fullerenol groups (*p* = 0.0153 and *p* = 0.0202, respectively; Tukey’s test; Figure 7D).

In the analysis of hippocampal *Cldn5* expression, a significant interaction effect between genotype and treatment was identified (F = 3.887, *p* = 0.0334, two-way ANOVA). Specifically, *Cldn5* expression was markedly reduced in the APP/PS1 vehicle group compared to the WT group (*p* = 0.0112, *p* < 0.05, Tukey’s test; Figure 7E), indicating a decrease in *Cldn5* levels in the APP/PS1 model. Furthermore, *Cldn5* expression was significantly diminished in the WT C_60_(OH)_24_-treated group relative to the WT vehicle group (*p* = 0.0102, *p* < 0.05, Tukey’s test; Figure 7E), suggesting that fullerenol treatment reduces *Cldn5* expression in WT mice. In the prefrontal cortex, a significant treatment effect was observed (F = 4.795, *p* = 0.0245, two-way ANOVA). Post hoc analysis indicated that *Cldn5* expression was lower in the C_60_(OH)_24_-treated group compared to the WT vehicle group (*p* = 0.0226, *p* < 0.05, Tukey’s test; Figure 7E). The data suggest a trend toward restored *Cldn5* expression levels in fullerenol-treated transgenic mice, although this effect did not reach statistical significance (Figure 7E). In the hippocampus, two-way ANOVA revealed no significant effect of genotype, treatment, or genotype × treatment interaction on *Bdnf* expression levels. However, post hoc analysis demonstrated that *Bdnf* expression was significantly lower in the WT C_60_-treated group compared to the WT vehicle group (*p* = 0.031, *p* < 0.05, Tukey’s test; Figure 7F), suggesting that fullerene treatment may reduce *Bdnf* levels. Similarly, the WT C_60_(OH)_24_-treated group exhibited a significant decrease in *Bdnf* expression compared to the WT vehicle group (*p* = 0.0092, *p* <0.01, Tukey’s test; Figure 7F), indicating a pronounced reduction in *Bdnf* levels following fullerenol treatment (Figure 7). These findings suggest that both C_60_ and C_60_(OH)_24_ treatments may negatively affect *Bdnf* expression in WT mice. In the prefrontal cortex, two-way ANOVA showed no significant effect of genotype, treatment, or genotype × treatment interaction on *Bdnf* expression levels. However, post hoc analysis revealed that *Bdnf* expression was significantly lower in APP/PS1 mice compared to WT mice (*p* = 0.0384, *p* < 0.05, Tukey’s test). C_60_ and C_60_(OH)_24_ treatments did not significantly affect *Bdnf* gene expression in the PFC. However, *Bdnf* expression in the C_60_(OH)_24_-treated group showed a trend toward restoration to WT levels, although this effect was not statistically significant (Figure 7F). Additionally, the expression levels of *Tubβ3* and *Sqstm1* genes were assessed, showing no significant changes across all studied groups (see Supplementary Figure S5).

## 4. Discussion

The present study examined the effects of prolonged administration of fullerene C_60_ and fullerenol C_60_(OH)_24_ on cognitive, emotional, histological, and molecular markers of AD using experimental conditions that closely mimic the disease, specifically aged female APP/PS1 mice. Our findings indicate that fullerenol administration, but not fullerene, significantly reduced amyloid plaque burden and prevented an increase in GFAP-positive cells in the cortex and hippocampus of mutants. Both treatments ameliorated molecular markers of neuroimmune abnormalities, specifically *Il1β* and *Sirt1*, in a region-specific manner and improved cognitive and behavioral outcomes, albeit in distinct ways. In vitro, fullerenol was non-toxic across a range of concentrations and protected against Aβ42-induced ROS generation in brain endothelial cells and astrocytes.

Our findings suggest that while both treatments influenced several AD-related parameters, only fullerenol appeared to reduce a key pathomorphological hallmark of AD, such as amyloid load. This may explain why APP/PS1 mice treated with fullerenol also exhibited greater positive changes in the investigated molecular markers of neuroinflammation and brain plasticity. One of the most notable findings was the differential effect of fullerene and fullerenol on amyloid plaque deposition. Congo red staining revealed that while fullerene treatment did not significantly alter plaque load, fullerenol reduced the maximum plaque size in the cortex and decreased the density of small plaques (<100 μm^2^) and the total density of plaques in the hippocampus and thalamus. This suggests that fullerenol may influence amyloid aggregation or clearance mechanisms, potentially due to its hydrophilic nature and enhanced reactivity compared to pristine fullerene.

Indeed, fullerenol primarily reduced plaque density without a significant change in the total plaque area. This suggests that fullerenol can alter plaque composition or distribution rather than significantly decrease the total plaque burden, highlighting its potential to improve cognitive function through mechanisms other than a direct reduction in Aβ deposition. For instance, it can be hypothesized that the administration of fullerenol might interfere with the aggregation process of Aβ peptides, leading to a reduction in the number of smaller or densely packed plaques (leading to a decrease in plaque density) without necessarily dissolving existing larger plaques, thus leaving the total plaque area relatively unchanged.

In addition, the administration of fullerenol can alter plaque composition. This treatment may modify the composition or structural properties of Aβ plaques, reducing their density or making them more diffuse. This can reduce plaque density but does not necessarily shrink the overall size of the plaques. Potentially, the administration of fullerenol may promote clearance mechanisms targeting soluble Aβ or smaller aggregates, decreasing overall plaque density but not affecting mature, larger plaques significantly. These effects of fullerenol can be attributed to its antioxidant and anti-inflammatory properties, which indirectly diminish plaque formation and density by modulating microglial activity and Aβ metabolism.

Strong associations between GFAP expression and amyloid pathology have been observed across cortical and hippocampal regions [40]. The persistent activation of astrocytes may ultimately be detrimental and exacerbate pathology and inflammatory signaling [41]. The results of the present study suggest that the long-term administration of fullerenol may attenuate astrocyte GFAP reactivity, which is a potential therapeutic target in AD. This decrease corresponds to a marked reduction in amyloid plaques within these regions, suggesting a therapeutic effect of fullerenol on neuroinflammation and plaque pathology.

In our study, long-term, low-dose oral administration of both fullerene and fullerenol exhibited neuroprotective and anti-inflammatory effects, albeit with slightly differential responses. For instance, behavioral assessments demonstrated that in the marble test, which assesses hippocampus-dependent cognitive function, fullerene-treated mutants displayed a significant increase in exploratory behavior, whereas fullerenol-treated mutants did not exhibit the same level of enhancement. However, in the object recognition test, which evaluates memory and cognitive flexibility, fullerenol-treated mutants demonstrated a significant improvement in the discrimination index compared to non-treated APP/PS1 mice, suggesting its beneficial effects on memory function. This indicates that while fullerene may have a more pronounced influence on general exploratory and novelty-seeking behavior, fullerenol appears to specifically target memory-related processes. The expression of inflammatory and neuroprotective markers further supports the differential effects of the two compounds. In the prefrontal cortex, fullerenol treatment significantly reduced expression of *Il1β* in APP/PS1 mice compared to untreated transgenic controls, suggesting a potential role in mitigating neuroinflammation. In contrast, C_60_ treatment did not significantly reduce *Il1β* expression. However, both fullerene and fullerenol increased *Il1β* expression in transgenic mice in the hippocampus, indicating a region-specific immune response. This finding is particularly relevant given that IL-1β is chronically elevated in AD brains and plays a key role in neuroinflammation [42,43]. IL-1β influences AD pathogenesis through its effects on APP expression, neurogenesis, and tau phosphorylation, contributing to disease progression through multiple signaling pathways [43,44]. These results suggest that while administration of fullerenol may help modulate inflammation in the prefrontal cortex, its effects in the hippocampus may be more complex, potentially exacerbating inflammatory responses in this region.

*Sirt1* expression, which is associated with longevity and neuroprotection [45,46], was significantly elevated in the hippocampi of APP/PS1 mice treated with fullerene and fullerenol, indicating a potential restorative effect in AD models. Conversely, in wild-type mice, both compounds led to a reduction in *Bdnf* expression, suggesting possible implications for neuroplasticity [47]. *Cldn5* expression, a marker of blood-brain barrier function [48], was notably decreased in wild-type mice treated with fullerenol in the hippocampus. However, in the prefrontal cortex, fullerenol showed a trend towards restoring *Cldn5* expression in APP/PS1 mice, although this effect did not reach statistical significance.

Thus, fullerenol appears to be more effective than fullerene in mitigating neuroinflammation and Aβ aggregation. These findings are consistent with previous studies that suggest hydroxylated fullerenes possess neuroprotective properties by modulating oxidative stress and inflammatory responses in the brain, potentially due to their ability to cross the blood-brain barrier [20]. In contrast, earlier studies have reported no detectable levels of fullerene C_60_ in brain tissue [18], implying that its neuroprotective effects are primarily mediated through systemic oxidative stress reduction rather than direct brain accumulation [3,4]. Consequently, the behavioral and molecular effects of chronic dosing with fullerene and fullerenol resulted in distinct outcomes, with the beneficial effects of the latter compound being seemingly more pronounced.

The differences in the effects of fullerene and fullerenol observed in our study pertain to the histological hallmarks of Alzheimer’s disease and, to a lesser extent, behavioral and molecular changes in the mutants. This may arise from the putatively different mechanisms of action of these compounds, where fullerenol likely acts directly on the brain by crossing the blood-brain barrier [20], whereas fullerene’s mechanisms of action may involve peripheral pathways via the gut microbiome, as recently demonstrated [17]. In the MPTP model of Parkinson’s disease, a 12-day regimen of fullerene C_60_ dissolved in olive oil at a concentration of 6.5 mg/kg primarily affected the gastrointestinal tract, regulating the gut microbiome, enhancing the production of short-chain fatty acids (SCFA), and normalizing gut barrier function [17]. This was accompanied by the normalization of motor function in the pole and rotarod tests, as well as normalized dopamine metabolism in the midbrain and striatum, preventing neuronal loss and mitochondrial damage in the substantia nigra and striatum [17].

Overall, the current demonstration of the efficacy of chronic administration of fullerene and fullerenol in mitigating Alzheimer’s disease-related pathology aligns with previous findings. As previously discussed, Kotelnicova et al. reported that water-soluble fullerenes inhibited monoamine oxidase B activity and reduced malondialdehyde production in mouse brain homogenates under oxidative stress conditions. Furthermore, they positively modulated α-amino-3-hydroxy-5-methyl-4-isoxazolepropionic acid receptor function in rat Purkinje neurons during patch-clamp experiments and enhanced spatial memory in C57BL/6 mice in an object recognition test [8]. In a separate study, unmodified pristine fullerene significantly reversed memory impairment in scopolamine-treated rats [6]. Vorobyov et al. demonstrated that pretreatment with hydrated fullerene restored disrupted hippocampal theta and cortical beta EEG rhythms caused by Aβ42 infusion in rats, potentially through presynaptic dopamine receptor involvement [9]. Similarly, Gordon et al. found that intrahippocampal Aβ injection in male Wistar rats resulted in memory deficits and damage primarily along the tri-synaptic hippocampal pathway, while pretreatment with hydrated fullerene C_60_ preserved neuronal structure and cognitive function, suggesting a neuroprotective effect [10]. Dai et al. showed that C_60_ treatment enhanced the synaptic localization of phosphorylated CaMKIIα in both Aβ42-treated cells and APP/PS1 mice, leading to improved spatial learning and memory [7]. Additionally, Perovic et al. demonstrated that treatment with C_60_(OH)_24–45_ significantly reduced cortical plaque accumulation in 5XFAD mice [6]. In a study by Kong et al., intraperitoneal injection of fullerenol (10 mg/kg for two days) reversed LPS-induced depression-like behaviors in mice. Moreover, fullerenol pretreatment significantly suppressed the LPS-induced upregulation of pro-inflammatory cytokines IL-6, IL-1β, and TNF-α in the hippocampus, reduced microglial activation, and restored neurogenesis impaired by LPS exposure in the dentate gyrus [15].

One of the fundamental mechanisms implicated in the pathogenesis of Alzheimer’s disease is the oxidative stress induced by Aβ in the brain [49]. Numerous studies have demonstrated that fullerene and its derivatives exhibit significant antioxidant activity both in vitro and in vivo [13,14,50,51,52,53]. These compounds exert potent antioxidant effects by scavenging free radicals, including hydroxyl, superoxide, peroxyl, and nitric oxide radicals, through mechanisms such as radical recombination and superoxide dismutase-like activity [54]. Notably, polyhydroxylated C_60_ derivatives have been shown to effectively mitigate excitotoxic neuronal death and exhibit high catalytic efficiency for superoxide neutralization, while also preventing lipid peroxidation and modulating enzyme activity across various systems [13,55]. Consistent with these findings, our study demonstrated that fullerenol C_60_(OH)_24_ effectively attenuated Aβ-induced ROS generation in mouse brain endothelial cells and astrocytes, corroborating previous studies that emphasize the compound’s capacity to reduce ROS overproduction in diverse cell types [53,56]. However, fullerenol failed to attenuate Aβ42-induced ROS generation in microglial cells and exhibited mild cytotoxicity at elevated concentrations. This divergent response likely reflects the distinct reactivity of microglia to external stimuli, consistent with their specialized immune function compared to astrocytes and endothelial cells. Notably, while the antioxidative properties of C_60_ fullerene have been demonstrated to mitigate neuroinflammation and protect dopaminergic neurons in MPTP-induced Parkinson’s disease models [17], its poor aqueous solubility prevented direct in vitro evaluation in our experimental system. This inherent limitation constrained our ability to compare its cellular efficacy with water-soluble derivatives in cell-based assays. Furthermore, emerging evidence suggests that impaired hepatic amyloid-beta degradation and inflammatory manifestations associated with gut dysbiosis in Alzheimer’s disease may contribute to Aβ accumulation in the brain [57,58,59]. Given that both C_60_ and C_60_(OH)_24_ preferentially accumulate in the liver following oral administration [18,25,60,61,62] and exert their primary effects in the gastrointestinal tract, where they can modulate the gut microbiome [17], prolonged use of these compounds may offer indirect neuroprotection. This effect could be mediated through the modulation of the gut microbiome [63,64,65,66,67,68,69] and inhibition of Aβ aggregation in the liver, potentially influencing neurodegenerative processes [57,70,71]. However, further research is required to elucidate the precise mechanisms underlying these effects and to explore their therapeutic potential in neurodegenerative diseases.

## 5. Conclusions

Our findings demonstrate the efficacy of chronic administration of fullerene or fullerenol on AD-related outcomes in aged APP/PS1 female mice, underscoring the potential of fullerenol as a therapeutic agent for AD due to its superior ability to reduce amyloid plaque burden compared to fullerene. While fullerenol administration appeared to enhance memory function in APP/PS1 mice, fullerene primarily induced behavioral invigoration in the tests used. These differential effects suggest that hydroxylation enhances the neuroprotective properties of fullerenes, likely by increasing bioavailability and reactivity. Future research should focus on elucidating the precise molecular mechanisms underlying these effects, particularly the influence of fullerene and its derivatives on oxidative stress pathways and amyloid metabolism [72,73,74,75,76]. Additionally, long-term studies assessing the safety and efficacy of fullerene and fullerenol in aged APP/PS1 mice could provide deeper insights into their therapeutic potential. Understanding the pharmacokinetics and biodistribution of these compounds will be crucial for translating these findings into potential clinical applications for AD treatment [77,78].

## Data Availability

Data available on reasonable request. To access data, Tatyana Strekalova (tatslova@gmail.com) and Sholpan Askarova (shaskarova@nu.edu.kz) should be contacted.

## Supplementary Materials

The following supporting information can be downloaded at: https://www.mdpi.com/article/10.3390/antiox14070834/s1. Figure S1: Experimental design; Table S1: The sequences of designed primers; Figure S2: Effects of APP/PS1 mutation and treatment with fullerene compounds on memory in Conditioned Taste Aversion; Figure S3: Effects of APP/PS1 mutation and treatment with fullerene compounds on emotionality; Figure S4: The proportion of various-sized plaques across the cortex, hippocampus, and thalamus and quartiles of plaque size distribution; Figure S5: Relative mRNA expression of Tubβ3 and SQSTM1 in the prefrontal cortex (PFC) and hippocampus (HIP) of transgenic (APP/PS) and wild-type (WT) mice.

## Abbreviations

The following abbreviations are used in this manuscript:

AD	Alzheimer’s disease
APP/PS1	APPswe/PS1E9 mice
WT	Wild-type
ROS	Reactive oxygen species
Aβ	Amyloid-beta peptide
EEG	Electroencephalogram
BBB	Blood-brain barrier
PD	Parkinson’s disease
DMD	Discrete molecular dynamics
ThT	Thioflavin-T
TEM	Transmission electron microscopy
FTIR	Fourier transform infrared
HFIP	1,1,1,3,3,3-hexafluoro-2-propanol
DMSO	Dimethyl sulfoxide
DMEM	Dulbecco’s Modified Eagle Medium
PBS	Phosphate-buffered saline
T-PMT	transmitted light photomultiplier
ROI	Region of interest
Il1β	Interleukin 1 beta
Il6	Interleukin 6
Gdf15	Growth Differentiation Factor 15
Sirt1	Sirtuin 1
Cldn5	Claudin 5
Bdnf	Brain-Derived Neurotrophic Factor
LPS	Lipopolysaccharide
TNF-α	Tumor Necrosis Factor alpha
PFC	Prefrontal cortex
MPTP	1-methyl-4-phenyl-1,2,3,6-tetrahydropyridine
SCFA	Short-chain fatty acids
