# Supplementary material for: Distinctive Effects of Fullerene C60 and Fullerenol C60(OH)24 Nanoparticles on Histological, Molecular and Behavioral Hallmarks of Alzheimer’s Disease in APPswe/PS1E9 Mice
Source: Antioxidants (Basel). 2025 Jul 8;14(7):834. doi: 10.3390/antiox14070834 (PMC12292005; doi:10.3390/antiox14070834)
Supplement: Supplementary file 1 [file antioxidants-14-00834-s001.zip › antioxidants-3622345-supplementary.pdf]

Supplementary file

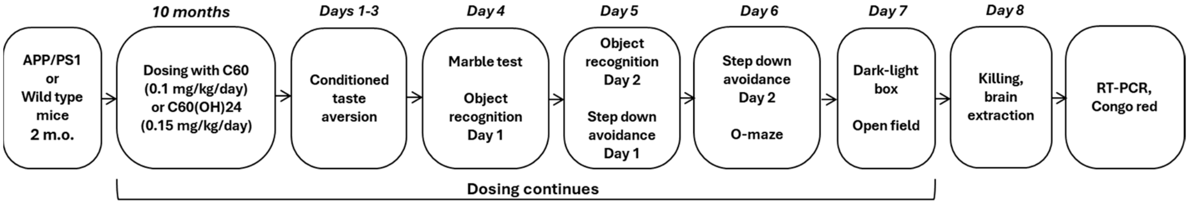

Figure S1. Experimental Design. After a ten-month regimen of administering fullerene (0.1 mg/kg/day) or fullerenole (0.15 mg/kg/day), mice underwent a series of behavioral assessments over a period of seven days. On the eighth day, the mice were euthanized, and their brains were dissected for subsequent RNA isolation and RT-qPCR (quantitative reverse transcription polymerase chain reaction) analysis, histological examination of amyloid plaques using Congo Red staining.

Table S1. The sequences of designed primers

№	Gene	Primer sequence 5′–3′	
1	**GAPDH**	For	ATGACCACAGTCCATGCCATC
		Rev	GAGCTTCCCGTTCAGCTCTG
2	**Interleukin-1 (IL-1)**	For	TTGAAGTTGACGGACCCCAA
		Rev	ATGTGCTGCTGCGAGATTTG
3	**IL-6**	For	TAGTCCTTCCTACCCCAATTTCC
		Rev	TTGGTCCTTAGCCACTCCTTC
4	**Tubulin-3Beta**	For	CGAGACCTACTGCATCGACA
		Rev	CATTGAGCTGACCAGGGA AT
5	**GDF15**	For	GACTGTGCAGGCAACTCTTG
		Rev	CGATACAGGTGGGGACACTC
6	**Sirtuin1**	For	TTGCAACAGCATCTTGCCTG
		Rev	CCTAGGGCACCGAGGAACTA
7	**Claudin-5**	For	GAGTTCAGCTTCCCGGTCAA
		Rev	CTCCCGCCCTTAGACATAGTTC
8	**SQSTM**	For	ATGGTGCACCCCAATGTGAT
		Rev	CTGCACAGGTCGTAGTCTGG
9	**BDNF**	For	CGGCGCCCATGAAAGAAGTA
		Rev	AGACCTCTCGAACCTGCCCT

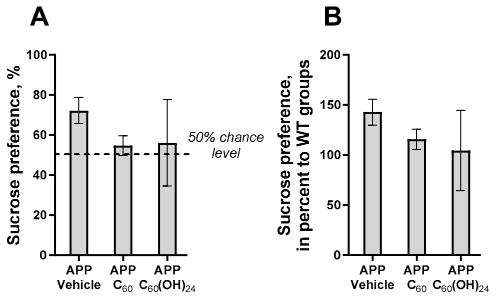

Figure S2. Impact of APP/PS1 mutation and treatment with fullerene or fullerenol on associative memory acquisition in the conditioned taste aversion paradigm. No significant differences were observed in the sucrose preference of mutant mice following LiCl injection, as indicated by (A) absolute values and (B) percentages relative to the respective WT groups. WT denotes wild types. Statistical analyses were conducted using two-way and one-way ANOVA, as well as one-sample t-tests. * indicates p<0.05, as determined by post hoc Tukey's test. # indicates p<0.05, as determined by one-sample t-test.

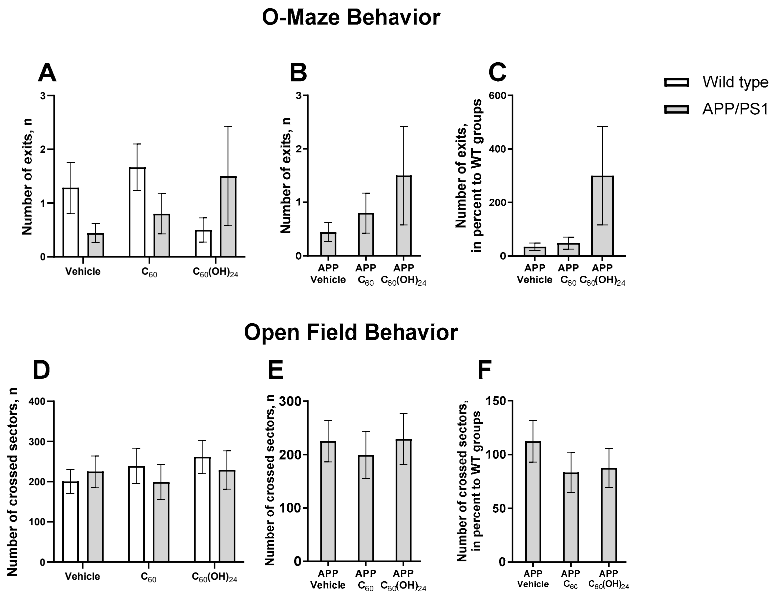

Figure S3. Alterations in emotionality in APP/PS1 mice subjected to treatment with fullerene or fullerenol. In the O-maze, no statistically significant differences were observed between groups in (A) the number of exits to the open arms, as well as in this measure among mutants in (B) absolute values and (C) percentage relative to the respective WT groups. In the open field test, no significant differences were detected between groups in (D) the total number of crossed sectors, as well as in this measure among mutants in (E) absolute values and (F) percentage relative to the respective WT groups. WT denotes wild types. Statistical analyses were conducted using two-way and one-way ANOVA.

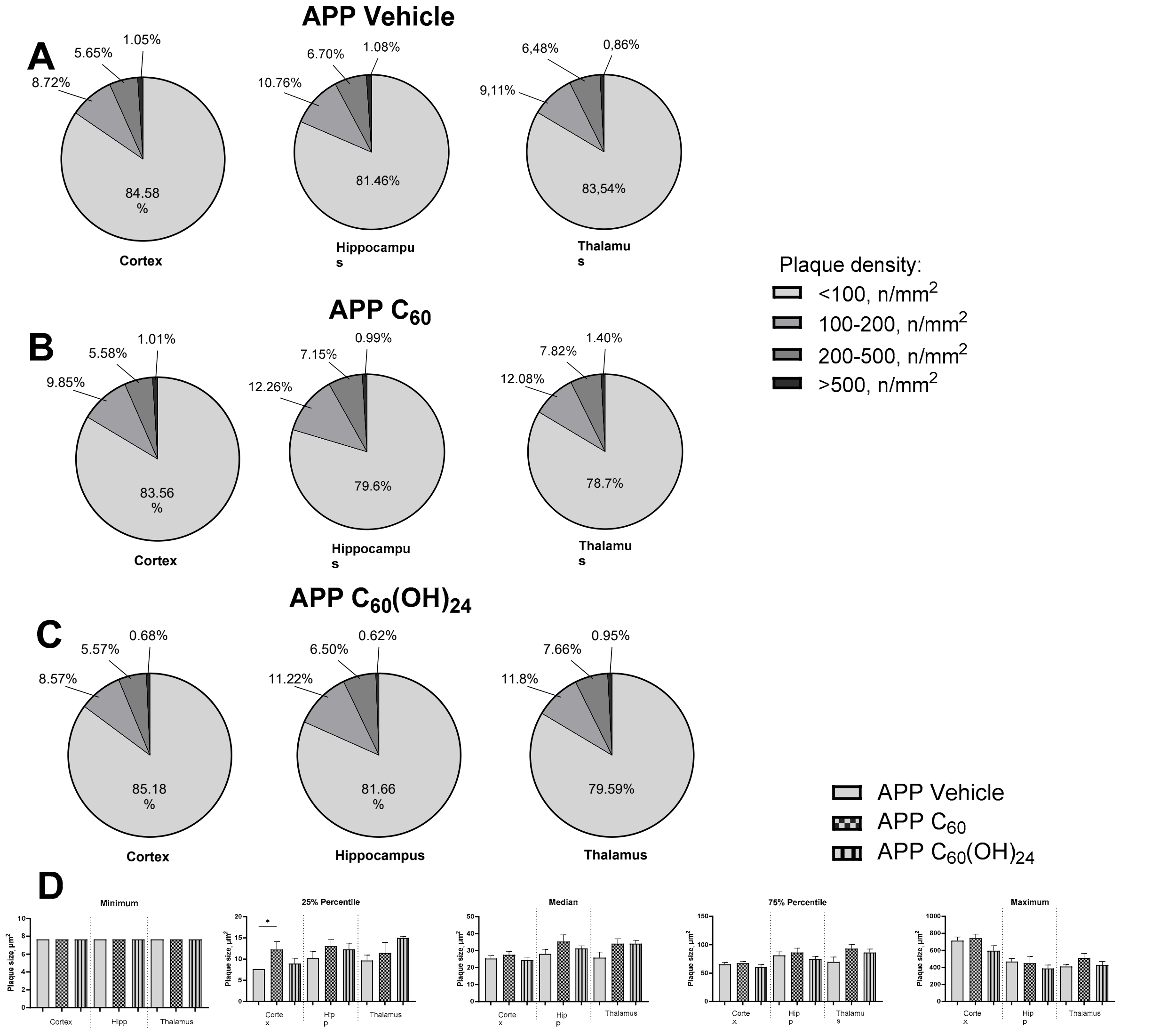

Figure S4. The proportion of plaques of varying sizes across the cortex, hippocampus, and thalamus, as well as the quartiles of plaque size distribution in APP PS1 mice treated with fullerene or fullerenol. The relative distribution of amyloid plaques of different sizes was consistent across the cortex, hippocampus, and thalamus in the (A) APP Vehicle, (B) APP C_{60}, and (C) APP $C_{60}(OH)_{24}$ groups. Notably, (D) the size of plaques in the cortex at the 25th percentile was significantly increased in the APP C_{60} group compared to the APP Vehicle group, while all other quartiles remained unchanged in the cortex, hippocampus, and thalamus. Statistical analysis was conducted using the Kruskal-Wallis test followed by post hoc Dunn’s test, $^{*}p<0.05$.

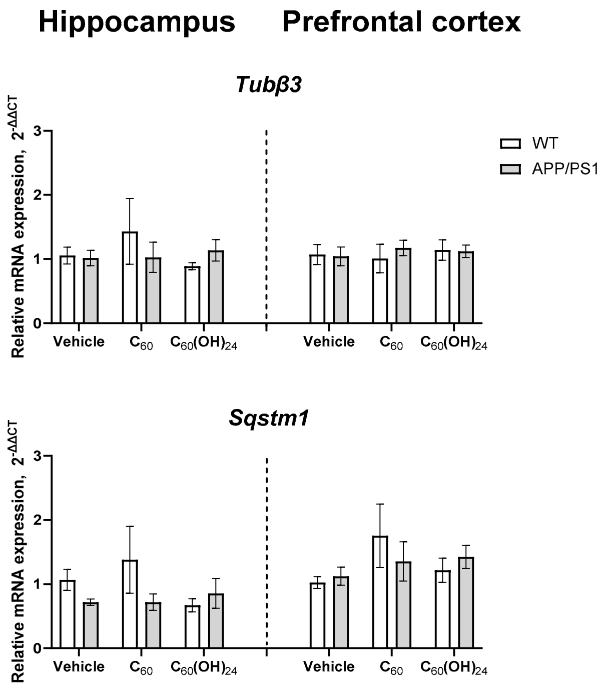

Figure S5. Relative mRNA expression of Tubβ3 and SQSTM1 in the brain of APP/PS and wild-type treated with fullerene or fullerenol. There were no significant changes in gene expression in of *Tubβ3* and *Sqstm1* found in the hppocampus and prefrontal cortex of expreimental groups ($p>0.05$, see ms text).
